# Supplementary material for: Gut–Kidney Axis on Chip for Studying Effects of Antibiotics on Risk of Hemolytic Uremic Syndrome by Shiga Toxin-Producing Escherichia coli
Source: Toxins (Basel). 2021 Nov 2;13(11):775. doi: 10.3390/toxins13110775 (PMC8622205; doi:10.3390/toxins13110775)
Supplement: Supplementary file 1 [file toxins-13-00775-s001.zip › toxins-1362035-supplementary.pdf]

Supplementary Materials: Gut–Kidney Axis on Chip for Studying Effects of Antibiotics on Risk of Hemolytic Uremic Syndrome by Shiga Toxin-Producing *Escherichia coli*

Yugyeong Lee, Min-Hyeok Kim, David Rodrigues Alves, Sejoong Kim, Luke P. Lee, Jong Hwan Sung and Sungsu Park

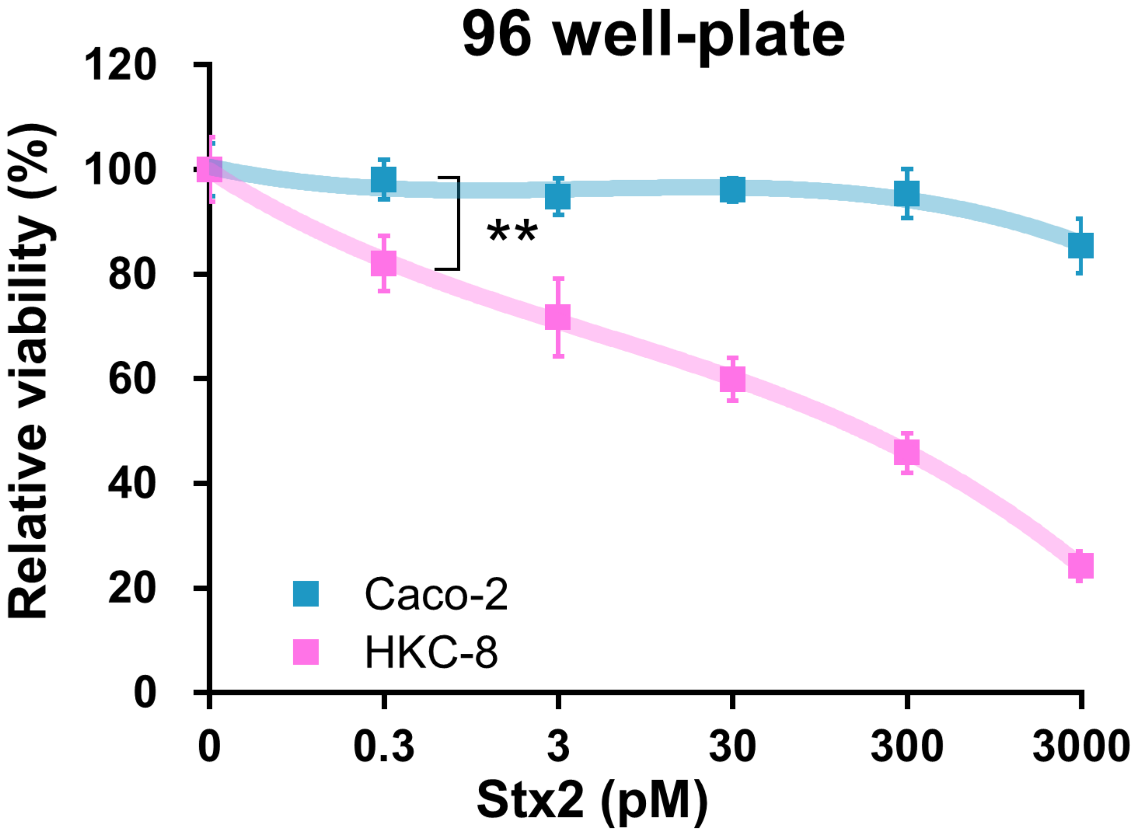

Figure S1. Viabilities of Caco-2 and HKC-8 cells treated with Stx2 at different concentrations (0-3 nM) in 96 well-plate for 72 h. Sample number (n) = 3, Student's *t*-test. ** $p < 0.01$.

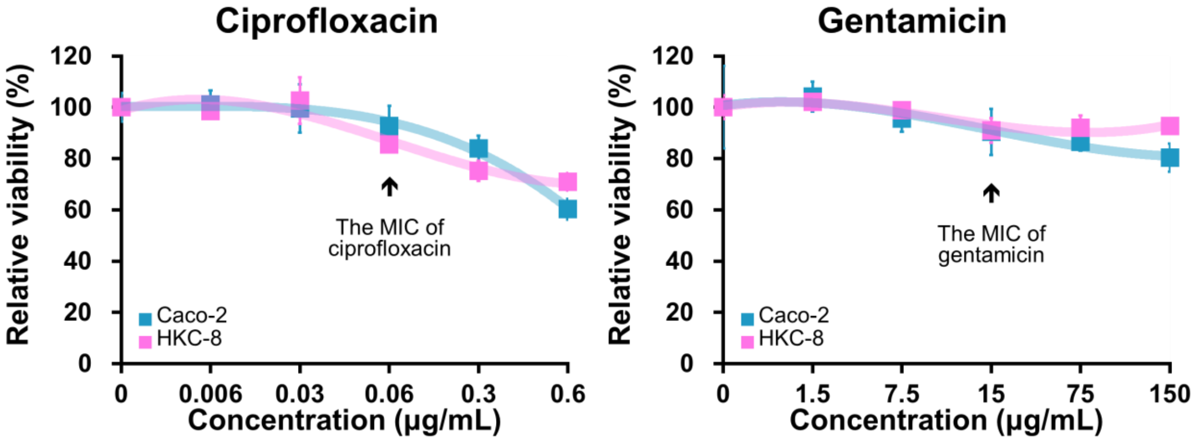

Figure S2. Viabilities of Caco-2 and HKC-8 cells treated with ciprofloxacin and gentamicin for 72 h.

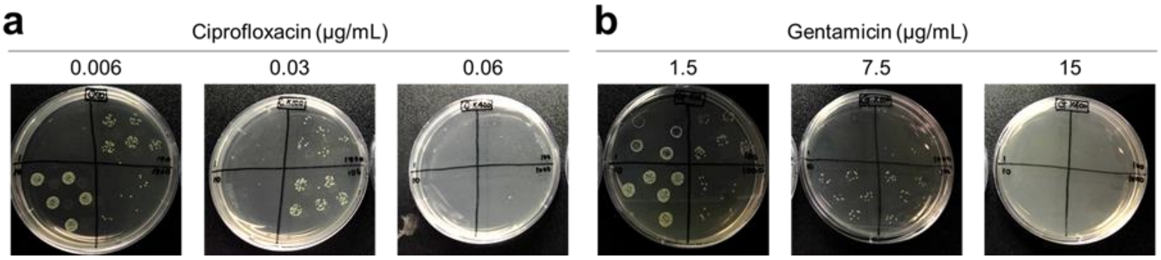

Figure S3. Bacterial colony counting assay. *E. coli* treated with either (a) ciprofloxacin (CIP) or (b) gentamicin (GEN) at various concentrations (0.006-0.6 μg/mL for ciprofloxacin; 1.5-15 μg/mL for gentamicin) overnight.

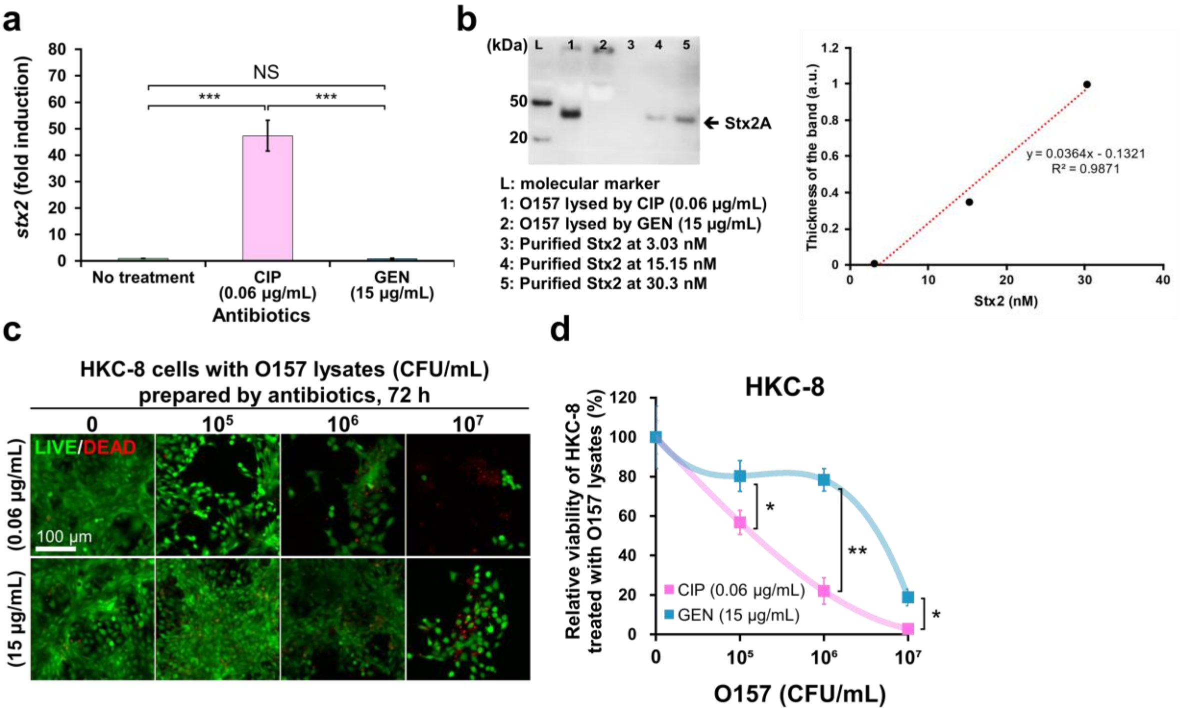

Figure S4. Effect of antibiotics on the expression of the *stx2* gene in O157 and cytotoxicity of O157 lysates on kidney cells cultured on a 24 well plate. (a) Expression of the *stx2* gene in O157 (10^5 CFU/mL) treated with CIP or GEN at their respective MIC for 2 h. All were normalized with GapA expression and given as relative to no treatment. (b) SDS-PAGE analyses of Stx2A in O157 (10^5 CFU/mL) treated with antibiotics (CIP and GEN) for 24 h and purified Stx2A at different concentrations (3.03-30.3 nM) and standard curve drawn by normalizing the thickness of bands by the thickness of lane 5. (c) LIVE/DEAD staining and (d) viability of HKC-8 cells in 24 well plate treated with lysates of O157 at different concentrations (10^5 - 10^7 CFU/mL) for 72 h. The lysates were prepared by CIP (0.06 µg/mL) or GEN (15 µg/mL) for 24 h before the treatments. (n = 3. Student's t-test. *$P < 0.05$. **$P < 0.01$, *** $p < 0.001$).

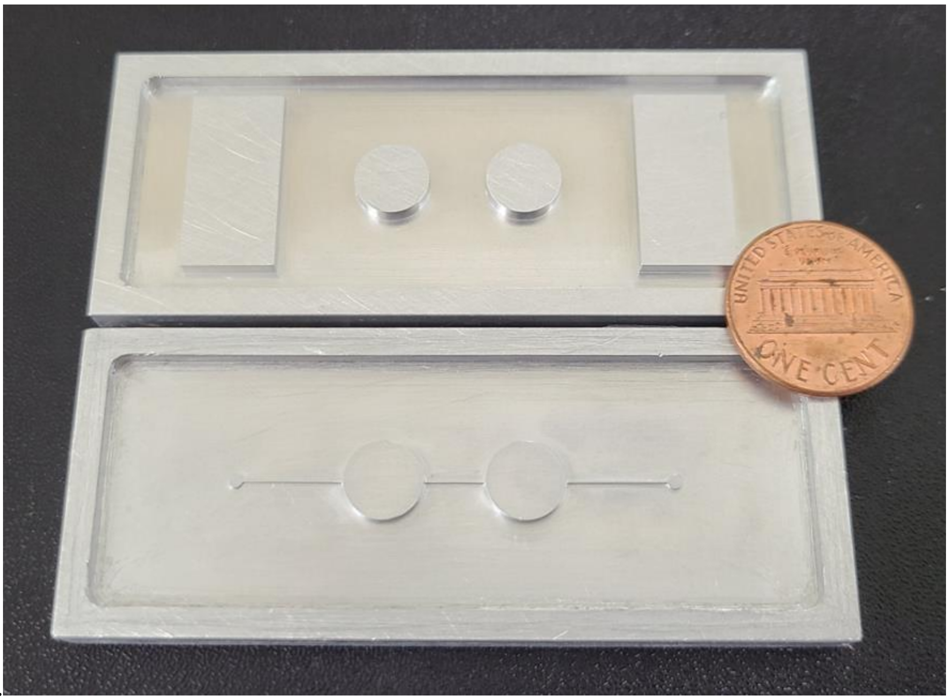

Figure S5. The molds for soft lithography of PDMS layers.

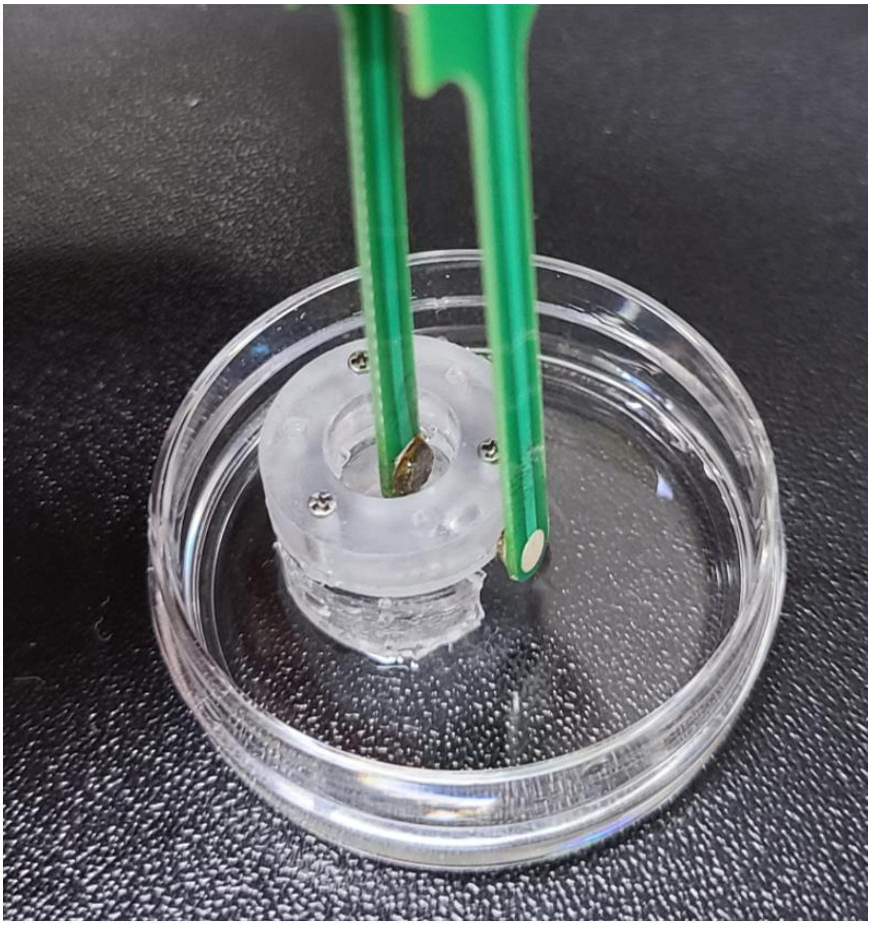

Figure S6. The usage of PDMS guide to make the TEER measurement easier and safer.

Materials and Methods

Real-time quantitative PCR (RT-qPCR) for stx2

To investigate the differential effect of CIP and GEN on the expression of the *stx2* gene, O157 at 10^7 CFU/mL was treated with either CIP or GEN at their respective MIC and incubated at 200 rpm and 37°C in the shaking incubator for 2 h. Since the mRNA coding for Stx2 is known to be induced during the early phase of treatment, the mRNA expression was quantified 2 h after each antibiotic treatment [34]. Total RNA was extracted from the treated bacteria using RNeasy mini kit from Qiagen (Venlo, Netherlands). The cDNA was synthesized from the total RNA using a RevertAid First Strand cDNA synthesis kit (Thermo Scientific; Waltham, MA, USA). Gene expression was quantified by RT-qPCR using iQ SYBR Green Supermix (Bio-Rad; Hercules, CA, USA) and a PCR machine, Lightcycler® Nano System, from Roche (Basel, Switzerland). Glyceraldehyde 3-phosphate dehydrogenase (GapA) was used to normalize the gene expression [31]. The primer sequences used were as follows: GapA: forward, 5′-GGTGCGAAGAAAGTGGTTATGAC-3′, reverse, 5′-GGCCAGCATATTTGTCGAAGTTAG-3′; Stx2: forward, 5′-GGGCAGTTATTTTGCTGTGGA-3′, reverse, 5′-TGTTGCCGTATTAACGAACCC-3′.

Methods for western blot

O157 at 10^5 CFU/mL was prepared and treated with either CIP or GEN at their respective MIC for 24 h. It was then filtered with a 0.2 μm filter 25CS020AS (ADVANTEC; Dublin, CA, USA) before separating its proteins with 10% SDS-PAGE and transferred onto nitrocellulose membrane. The membrane was then washed three times with 0.1% Tween-20 (Sigma-Aldrich) in Tris buffered saline (TBS-T) and blocked with 5% skim milk/TBS-T. After the washing step, it was incubated with rabbit anti-Stx2 antibody (#765L) from List Biological Lab, Inc. (Campbell, CA, USA) at a 1:2,000 dilution. It was washed three times with TBS-T and incubated with goat anti-rabbit IgG conjugated to horse radish peroxidase (HRP) from Seracare Life Sciences Inc. (Milford, MA, USA). It was finally developed using a Clarity Western ECL Substrate (Bio-Rad) and its luminescence was expo6sed to X-ray films (Agfa; Mortsel, Belgium).

References

34. Corogeanu, D.; Wilmes, R.; Wolke, M.; Plum, G.; Utermohlen, O.; Kronke, M. Therapeutic concentratio ns of antibiotics inhibit Shiga toxin release from enterohemorrhagic *E. coli* O104:H4 from the 2011 Germa n outbreak *BMC Microbiol.* **2012**, *12*, 160 doi:10.1186/1471-2180-12-160
